# Supplementary material for: Motor-Independent Cognitive Testing in Motor Degenerative Diseases
Source: J Clin Med. 2022 Feb 3;11(3):814. doi: 10.3390/jcm11030814 (PMC8836999; doi:10.3390/jcm11030814)
Supplement: Supplementary file 1 [file jcm-11-00814-s001.zip › jcm-1544513-supplement.pdf]

Motor Independent Cognitive Testing in Motor Degenerative Diseases

Henning Schmitz-Peiffer [1], Elisa Aust [1], Katharina Linse [1,2], Wolfgang Rueger [3], Markus Joos [3], Matthias Löhle [4,5], Alexander Storch [4,5,6] and Andreas Hermann [5,6,7,*]

Supplementary Materials

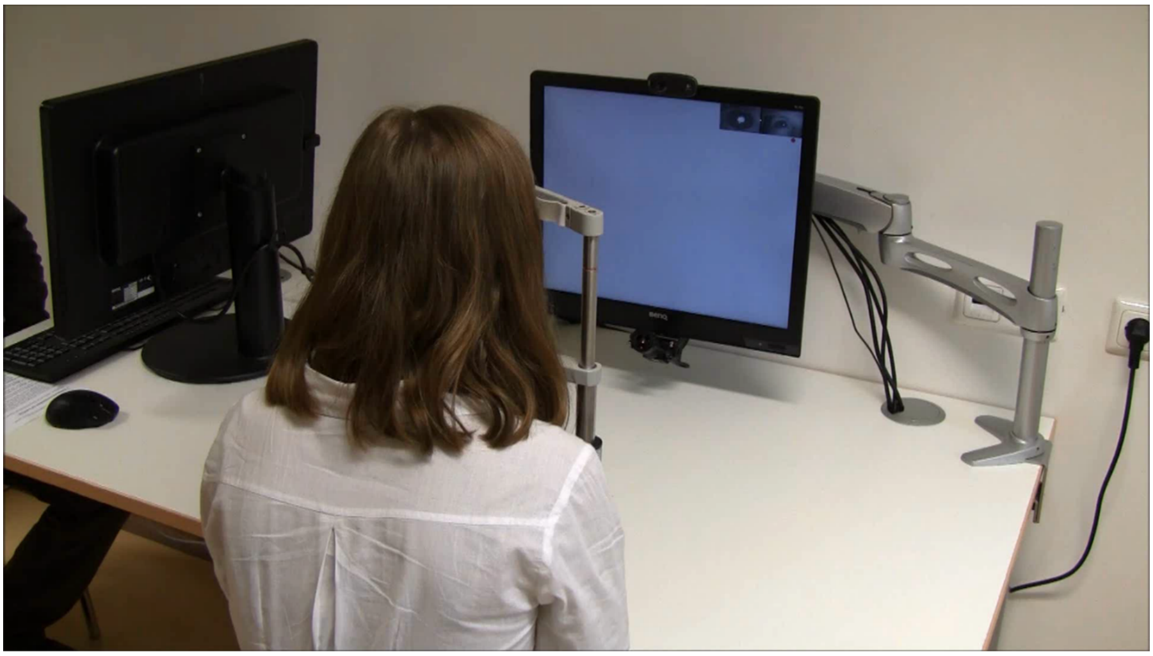

Figure S1. Setup for eTMT using a chin rest, used for mobile patients to control for involuntary head movements. The second monitor serves as an observer monitor to control for possible limitations of reliability or confounding factors, e.g., deficient calibration, technical issues, or subjects not reading the instructions.

Video S1. Calibration task.

Video S2. eTMT-B including practice task.
